# Building up an ecologically sustainable and socially desirable post-COVID-19 future

**DOI:** 10.1007/s11625-021-00940-z

**Published:** 2021-04-05

**Authors:** Rémi Duflot, Stefan Baumeister, Daniel Burgas, Kyle Eyvindson, María Triviño, Clemens Blattert, Anna Kuparinen, Mária Potterf

**Affiliations:** 1 School of Resource Wisdom, University of Jyvaskyla, Jyvaskyla, Finland; 2 Department of Biological and Environmental Sciences, University of Jyvaskyla, Jyvaskyla, Finland; 3 School of Business and Economics, University of Jyvaskyla, Jyvaskyla, Finland; 4 Natural Resource Institute Finland (LUKE), Laatokartanonkaari 9, Helsinki, Finland

**Keywords:** Anthropocene, Biodiversity loss, Climate change, Degrowth, Environmental justice, Environmental policy, Sustainability, Well-being

## Abstract

COVID-19 crisis has emphasized how poorly prepared humanity is to cope with global disasters. However, this crisis also offers a unique opportunity to move towards a more sustainable and equitable future. Here, we identify the underlying environmental, social, and economic chronic causes of the COVID-19 crisis. We argue in favour of a holistic view to initiate a socio-economic transition to improve the prospects for global sustainability and human well-being. Alternative approaches to “Business-As-Usual” for guiding the transition are already available for implementation. Yet, to ensure a successful and just transition, we need to change our priorities towards environmental integrity and well-being. This necessarily means environmental justice, a different worldview and a closer relationship with nature.

## Introduction

Humanity is struggling with the outbreak of a novel coronavirus disease, which has emerged in late 2019 (COVID-19), and the social and economic consequences resulting from the still ongoing worldwide pandemic. At the time of writing, it is reported that COVID-19 has occurred in 221 countries, infected over 113 million people and caused 2.5 million fatalities (Worldometers, 2021). Beyond health issues, the pandemic has caused a global economic recession that peaked in spring 2020, before restrictions loosened in the second semester. In total, the Gross Domestic Product (GDP) in 2020 fell by 4.9% in OECD countries (OECD 2021a), causing unemployment rate to jump from 5.2% to 6.9% over the same period (OECD 2021b). In the short-term, over one billion people may enter extreme poverty under the most extreme economic scenario, mainly in developing countries, but also in middle-income and developed countries (Sumner et al. 2020).

We considered what brought us to such global havoc. We assert that the current crisis is a prominent sign of a degraded biosphere and shows the lack of sustainability of the anthropogenic globalized system. The COVID-19 outbreak has emphasized that human societies are vulnerable, unequally equipped, and unprepared to cope with global disasters. The “COVID-19 crisis*"* serves as a full-scale crash test for our resilience towards an uncertain future, as we enter a new ecological norm under climate change, degraded ecosystems, and biodiversity loss.

To prepare ourselves to face these major threats to humanity, it is crucial to identify what lessons can be learnt from the COVID-19 crisis and how it affects Earth systems (Diffenbaugh et al. 2020; Manzanedo and Manning 2020). To prevent the further spread of COVID-19 and to lessen human deaths, large-scale and drastic decisions have been taken around the world, such as social distancing, lockdowns, curfews, quarantines, and border closures. Although highly variable in their application, severity and efficiency across different countries (Thu et al. 2020), these decisions provoked a dramatic and ad hoc change in the behaviour of millions of people in a short timeframe. We argue that today’s decisions should not only focus on prompt pandemic mitigation, but also address long-term adaptation to environmental changes, which are coming slowly but persistently. In this comment, we build a post-COVID-19 narrative based on scientific arguments on why and how to act for an urgently needed ecologically sustainable and just socio-economic transition.

## What has been highlighted by the crisis?

The emergence of zoonotic infections, i.e. diseases transmitted from animals to humans, such as SARS-COV1, MERS, NIPAH, and swine or bird influenza, has dramatically increased over the last decades (Wilcox and Gubler 2005). Deforestation and wildlife hunting facilitate the transmission of such diseases by bringing humans and associated domestic animals in closer contact with novel pathogens from wildlife (Keesing et al. 2010; di Marco et al. 2020). At the same time, deterioration of biodiversity decreases ecosystem functioning and reduces the protective effects of biodiversity against infectious diseases, suppressing regulation and dilution of pathogen reservoirs (Keesing et al. 2010). Intensive animal husbandry also promotes the appearance and further spread of these diseases, due to concentration and transport of animals prone to infections. COVID-19 is a new case of zoonosis that emerged after illegal trafficking and consumption of bushmeat, possibly of bats or pangolins (Lam et al. 2020). The current health and economic crisis resulting from COVID-19 underscores the risks of disregarding the importance of the link between human health and the state of the environment. Such interconnection between the health of humans, animals and ecosystems is recognized in the One Health approach that aims to link the biological and the social systems and is supported by a policy framework of international organizations (di Marco et al. 2020).

The COVID-19 pandemic, alike historical pandemics (Wade 2020), challenges the societal ability to provide comprehensive access to key services, and shows how poorly integrated some communities are. For instance, food or health systems suffer from workforce shortage and/or structural limitations, to the detriment of the most vulnerable communities (Willan et al. 2020; Laborde et al. 2020). Health care systems around the globe reached their capacity limits, even among the wealthiest countries (Sachs et al. 2020), demonstrating the value of publicly funded and universal health systems (Oliver 2020; Etienne et al. 2020). Severe restrictions to mobility (lockdown) to combat the progression of the pandemic has exacerbated existing social inequalities between and within countries (von Braun et al. 2020). For instance, remote schooling or work relies on proper internet access and suitable workspace, which is difficult to attain with limited economic resources. In addition, it is often easier for white-collar workers to work remotely compared with blue-collar workers that are more likely to lose their jobs due to halted manufacturing, or that must expose themselves to make a living. Similarly, COVID-19 enhanced gender disparities. More women than men have lost their jobs, occupy essential professions that expose them to infection and psychological stress, and have experienced work disruption due to increased responsibilities in childcare and domestic duties (Carli 2020). This means that low-income individuals, marginalized communities, and women are at the greatest risk and suffer more the economic, livelihood, or health consequences due to COVID-19.

In addition to environmental degradation and social inequalities, the economic recession due to COVID-19 highlights the risks associated with the hyper-connected economic and financial systems worldwide. Increased complexity, lack of diversification, strong interdependencies, and just-in-time supply chains generate systemic risks and instability (Helbing 2013), making such systems less resilient to unexpected events, such as the COVID-19 pandemic. Global connections mean less self-sufficiency and higher risk of disruptions. For example, economic functioning and free trade (usually associated with profitability and openness) promote the use of cheap, fast, and long-distance transportation, and can lead to the homogenization of tastes, products, and processes (Chu-Shore 2010). At the beginning of the crisis, the loss of accessibility to international trade with China—a single but major economic agent—deteriorated supply chains of industrial, medical, and key daily consumable goods worldwide (Luo and Tsang 2020). This cascading effect demonstrated the general risks associated with production relying on global connectivity.

## Consequences of COVID-19 crisis on sustainability

Although restricted mobility and economic activity have caused a dramatic improvement in global air quality (Venter et al. 2020) and sometimes resulted in cleaner water and reduced noise pollution, it is currently not clear what will be the final impact on climate change and biodiversity conservation (Corlett et al. 2020). First, although China already banned bushmeat consumption, the continuous demand might create black markets, while new regulation does not restrict medicinal use of wildlife products (Wang et al. 2020). Second, the boom in wildlife sightings during lockdowns (especially in cities) may be simply due to increased detection of species that were always there rather than true recolonization of spaces made available by reduced human activities (Zellmer et al. 2020). In any case, such an effect can be as short-lasting as the length of the lockdowns. Finally, conservation efforts of biodiversity hotspots, especially in developing countries, rely heavily on nature-based tourism that has collapsed, putting at odds conservation enforcement and value for local communities (Rondeau et al. 2020).

The drop in greenhouse gas emissions during the lockdowns—driven by reduced transportation and energy demand—drew much attention (Liu et al. 2020). Yet, the emissions observed during lockdown were comparable to those of 2006. Most importantly, the largest estimate of annual emission drop for 2020 (− 7.5%) represents a reduction rate needed every year over the next decade to limit climate change to a 1.5 °C warming (UNEP 2019; Le Quéré et al. 2020). Based on lessons learned from the 2008 financial crisis it is likely that the revitalization of economies with cash and tax exemptions, combined with current low prices of fossil fuels, will overcompensate the drop of greenhouse gas emissions in the coming years (Peters et al. 2012). Moreover, recent and future advances in conservation and sustainability policies can be put on hold, or even reversed, as policymakers might prioritize economy revitalization. For instance, in reaction to the closure of borders and dramatic drop in passenger numbers, the aviation industry has urged to postpone or revise the planned global policy measures aimed at reducing environmental impacts, e.g. the offsetting scheme of greenhouse gas emissions that was to start in January 2021 (Amankwah-Amoah 2020).

## Moving towards a sustainable future path

Although stimulus packages enforced by governments can represent opportunities to boost the development of green energies to mitigate climate change, they can also have adverse effects if misguided (Sovacool et al. 2020). Governments often aim financial support at incumbent industries with high employment, such as car manufacturers, airlines, and oil companies. We suggest that recovery funds could rather target financial support towards forward-looking objectives, supporting innovative technology that could mitigate climate warming and biodiversity loss (Wilson 2018; Kuokkanen et al. 2019). Accounting of the global recovery funds shows that even a fraction of the investments, if directed to the development of sustainable energy, could be sufficient to meet the Paris Agreement objectives (Andrijevic et al. 2020). However, the same study stresses that divestments and removal of subsidies towards fossil-fuel activities are also necessary, and international support is needed for developing countries. Additional examples include investments to stimulate sustainable productions, such as work-intensive organic farming, low-carbon transportation (e.g. upgrade railway lines), or recyclable materials (circular economy).

**Fig. 1:**
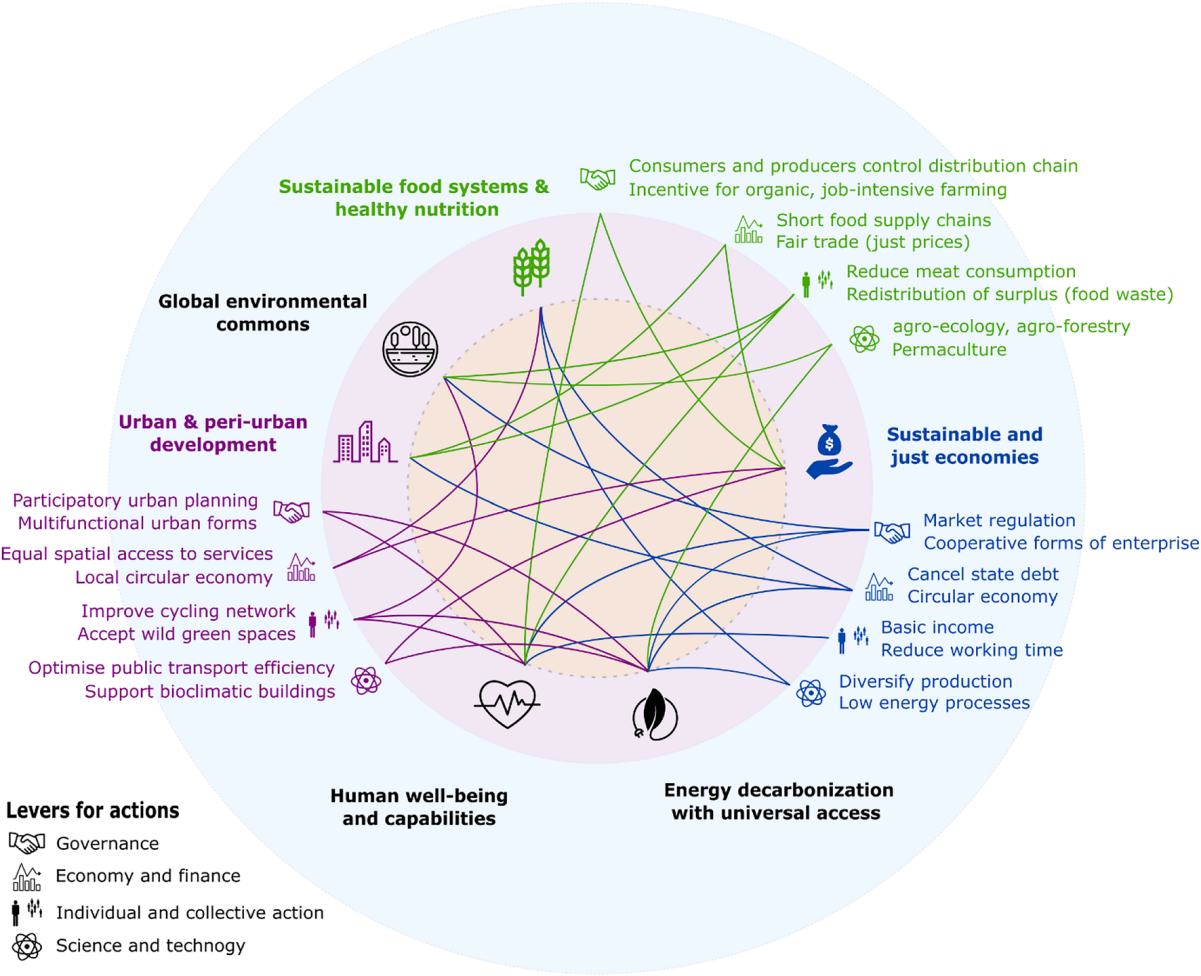
Selected examples of alternatives aiming to increase sustainability (outer circle) for three out of the six entry points for transformations (bold coloured text, intermediate circle) from the Global Sustainable Development Report 2019 (Messerli et al. 2019) and their positive interactions with other entry points (coloured links, inner circle). Credit for icons: Freepik, PixelPerfect, Geotatah, Google and GoodWare from www.flaticon.com

The 2030 Agenda for Sustainable Development (UN General Assembly 2015)—although still imperfect (Zeng et al. 2020)—provides a detailed roadmap to achieve the Sustainable Development Goals (SDGs). However, the Global Sustainable Development Report 2019 (Messerli et al. 2019) showed that under the overall trends prior the pandemic, not a single SDG will be achieved by 2030. The Sustainable Development Report 2020 estimated that the pandemic has worsen this tendency (Sachs et al. 2020). Here we echo the views of the authors of these reports and stress that there exist many sustainable socio-technical systems, i.e. systems involving the interaction of technology and human beings, which can serve as alternatives to the current organization of human activities. Most alternatives are ready to be implemented through sectorial entry points for transformation and can contribute to mitigate the social and economic consequences of the COVID-19 pandemic (see Fig. 1 for selected examples). A systemic approach is necessary as the multiple aspects of sustainable development are intrinsically interrelated. In other words, any specific action can and should contribute to several dimensions of sustainability (Fig. 1).

There are opportunities for mutually beneficial policies between the SDGs related to biodiversity conservation and food security, and the prevention of pandemics (di Marco et al. 2020). Indeed, preserving intact ecosystems and promoting sustainable land use can contribute to prevent the emergence of future infectious diseases. Specifically, policies that prevent tropical deforestation and limit wildlife trade will reduce the risk of future zoonosis outbreaks, contribute to biodiversity conservation and limit climate change; at considerably lower socio-economic costs compared to the COVID-19 crisis (Dobson et al. 2020).

## Prioritizing sustainable well-being

Despite numerous signatories of the United Nations agreement on sustainable development, the commitment of countries has been highly variable, and in many cases, it has not been translated into effective national policies (Sachs et al. 2020). Rather marginal environmental and social advances are made without compromising the main economic paradigm represented by free market, profitability and growth. Economic growth contributes to biodiversity loss and environmental degradation in general, as intensive use of resources and trade lead to climate and land-use change, and spread of invasive species (Otero et al. 2020). Despite recurrent claims on decoupling economic growth from natural resource extraction and CO_2_ emissions, e.g. through the use of more efficient technologies, it still has not happened, and it is unlikely that it will (Gómez-Baggethun 2020). Even most policy documents on sustainable development do not depart from the economic growth paradigm and barely mention decoupling and how it can be achieved (Otero et al. 2020).

We argue that the implementation of a sustainable policy (as described in previous section) requires a change in societal priorities, shifting from gross domestic product (GDP) to human well-being and a healthy environment (Spash 2020). Slow, zero or negative GDP growth is often wrongly associated with a loss of well-being (Otero et al. 2020; Gómez-Baggethun 2020). This assumption depicts a utilitarian philosophy singularly linking increased consumption with increased human well-being. However, human well-being is a multidimensional state, with no fixed definition (King et al. 2014). In social sciences, well-being is usually defined by several bio-physical and social components including material subsistence, security, physical and mental health, social and physical environmental conditions, social connections and relationships, education, and abilities for social participation (including political voice); leaving out the subjective perception of well-being. To quantify progress towards well-being, economic measures should account for such multidimensional perspective. For instance, the genuine progress index (GPI) offers a more comprehensive measure of economic benefits and costs compared to GDP by incorporating consumption, inequalities, social welfare, and environmental costs. As opposed to GDP, GPI has stagnated in many countries and even slightly decreased globally since the mid-1970s (Kubiszewski et al. 2013).

The GPI has been linked to the Sustainable Development Goals (SDGs) of the 2030 Agenda to create a set of indicators aimed at “*a prosperous, high quality of life that is equitably shared and sustainable*”(Costanza et al. 2016). The need of aggregating multiple components into a single-value indicator leads to some arbitrary inclusion and exclusion of indicators (Berik 2020). Therefore, these indicators are inherently imperfect in capturing the complexity of reality, and they should be viewed rather as a guide towards sustainable well-being and not as sustainable well-being itself. In particular, both GPI and the SDGs carry the risk of overemphasising socio-economic welfare to the detriment of environmental health (Kubiszewski et al. 2013; Zeng et al. 2020). Given this risk, we need to simultaneously consider multiple dimensions of sustainability and well-being and acknowledge the difficulty to find comparable measures for all components. There are also ethical problems in the monetization of non-market components, such as ecosystem services. Hence, looking at the different elements of GPI and SDGs may prove more useful than their combined value to evaluate current state and define proper policies. The COVID-19 pandemic has questioned and shed lights on what is important for human societies and what is not. Existing measurable objectives can be used to set new priorities and direct individual and collective decision-making.

## Environmental justice as a societal prospect

Implementing a fair and equitable transition towards a sustainable path that provides well-being for all is a challenging task. However, socio-economic systems are based on individual and collective decisions (at least in democracies), and they can be changed. The COVID-19 crisis has shown that governance plays a crucial role in the successful response to a major crisis and that structural and transformative changes need strong yet transparent political leadership and investments (Etienne et al. 2020). While more authoritarian approaches (e.g. in China) might yield stronger enforcement and better societal compliance, they also failed to take crucial initial action by suppressing the information on the disease emergence (Ang 2020). Meanwhile, democratic regimes had a harder time implementing forceful actions, but were in most cases able to maintain a better information flow and trust (Greer et al. 2020). Independently from regime type, compliance with public health measures and the quality of life during and after the pandemic are shaped by wise leadership, state capacity and pre-existing social policy (Ang 2020; Greer et al. 2020). For instance, some leaders (e.g. in USA or Brazil) have initially attempted to deny the actual problem, delaying implementation of adequate controlling measures and instigating confusion.

A sustainable transition, alike an adequate response to a pandemic, requires a cohesive and inclusive society where people adhere to collective actions. Justice (in a broad sense) should be seen as a precondition to create institutional trust and social security, and thus to encourage citizens’ participation in collective projects. As a long-term response to the COVID-19 crisis, a large array of regulations could be implemented by governments to address inequalities through social justice (Ashford et al. 2020; van Barneveld et al. 2020). Three aspects of justice are needed to ensure a just sustainable transition (McCauley and Heffron 2018).

First, *distributive justice* supposes that not only economic and well-being benefits are equally shared but so are the negative environmental consequences due to climate change or pollution. Thus, environmental and social common goods, such as air quality, water distribution, food system, education, health, and culture should be strongly regulated, supervised by citizen controls (e.g. through participation in decision boards or cooperative forms of enterprise), and largely remain out of markets (i.e. managed or closely supervised by public institutions). Such transparent and participative management should reduce the continuation of the “tragedy of the commons”. Second, *procedural justice* is a process to solve potential conflicts between concurrent interests. A multiscale democratic and participatory decision-making needs to be adopted to ensure that all citizens, communities and social groups are represented and have equal decision power. In this configuration, top-down international and national regulations must provide guidelines to highlight conservation requirements and limit potentially destructive activities, while bottom-up planning schemes, e.g. at the municipality level, would allow adaptation to the local environmental context and cultural preferences. Third, *restorative justice* repairs the harm done to an individual or a community. For instance, a great fear is that sustainable transition would increase unemployment by phasing out polluting industries. However, subsidy and tax exemption policies, as well as workers’ training, can gradually redirect economic activities towards less polluting and more sustainable sectors (such as organic farming or health and care services). This might, however, meet social and cultural resistance, as some communities tend to associate their identity with their professions (e.g. in industrial towns). Reducing work hours is another option to limit unemployment due to the sustainable transition, and so is the introduction of a universal basic income that provides citizens with more freedom and less dependence on paid work (Ashford et al. 2020).

Beyond justice, a sustainable transition also relies on a shift in social representations (i.e. systems of shared values allowing societal behaviour and communication) and human–nature relationship across most cultures. Along with socio-economic development, societies globally have become more individualistic (Santos et al. 2017), making catastrophes more likely to occur, more damaging, and more likely to spread than in societies with a higher sense of mutual care for common goods and collaboration. At the same time, the lifestyle of the wealthiest part of population shows unsustainable consumption patterns to distinguish themselves from the lower class, influencing and pushing up the consumption of the growing middle class (Otto et al. 2019). Moreover, the increasing sense of detachment of people from the biosphere (to the extent of experiencing *biophobia*) makes it more difficult for individuals to spend time and resources in caring for nature (Pyle 2003). A sustainability transition requires a shift in worldview from the technological utopia of modernism (Gómez-Baggethun 2020) to the realization of humanity's dependence on ecosystems, leading to a reconnection with nature. Potential directions to encourage such evolution of worldviews include promotion of the philosophy of minimal ownership, ethics of land integrity, and nature-centred education (Pyle 2003).

## Conclusion

In the immediate aftermath of the COVID-19 crisis, we must document its impact on individuals, communities, and societies; allowing an opportunity to clarify and eventually change our shared planetary direction. As such, we must proactively adapt to the potentially harder times ahead of us aggravated by climate change, ecosystem degradation, and biodiversity loss. The COVID-19 crisis offers a unique opportunity to move towards a greener, more sustainable and equitable society to avoid the destruction of our planet and our own well-being. Many alternative futures exist to live well under the new ecological norm. To make it happen, we shall reorganize our worldview and re-invent governance and the role of citizens at multiple scales. It is an arduous effort, only possible with vast cooperation between societies, and the shared conviction that mutual benefits arising from collaboration might exceed those arising from individualistic competition.
